# Facing up to programmatic challenges created by the HIV/AIDS epidemic in sub-Saharan Africa

**DOI:** 10.1186/1758-2652-14-S1-S1

**Published:** 2011-07-06

**Authors:** Shirin Heidari, Anthony D Harries, Rony Zachariah

**Affiliations:** 1 International AIDS Society, Geneva, Switzerland; 2 International Union Against Tuberculosis and Lung Diseases, Paris, France; 3 London School of Hygiene and Tropical Medicine, London, UK; 4 Médecins sans Frontières, Operational Centre Brussels, Medical Department, MSF- Luxembourg, Luxembourg

## Abstract

Three decades after the emergence of HIV, we have made great strides in our response to the epidemic, from prevention of transmission to testing and treatment. However, it is still common in high-prevalence settings for people to not know their HIV status, and estimates are that globally, a mere 36% of those eligible for treatment are receiving it. On top of this, for every person with HIV entering treatment, two more are infected. The operationa obstacles to overcoming the challenges and fully implementing proven strategies are numerous. The operational research and implementation sciences aim to provide a sound basis for how to maximize the use of limited resources by investigating the best models to deliver services and implement programmes in various settings and contexts. In this special issue, the *Journal of the International AIDS Society* intends to highlight some of the operational and programmatic challenges that are faced in sub-Saharan Africa, home to the largest population living with HIV. Our hope is that readers gain insight into some of the challenges associated with the HIV/AIDS epidemic and a changing environment in the region, and become familiar with some applications of operational research and implementation science in HIV healthcare settings.

## 

Three decades into the HIV epidemic, we have made remarkable progress. We have sensitive and specific tests to detect infection; there are effective antiretroviral (ARV) drugs to prevent the virus from continuing its relentless onslaught on the body’s immune system; we can reliably measure treatment success using techniques that can determine the level of immunosuppression or quantify viral load in plasma; available resistance genotyping allows us to detect the emergence of strains insensitive to prescribed drugs; and there are guidelines to help clinicians switch patients to more effective anti-retroviral combinations.

We also have a relatively sound understanding of how to prevent HIV transmission and acquisition; effective suppression of viral load in patients is believed to minimize the risk of HIV transmission, and there is a strong conviction that treatment can have a significant impact on curbing the epidemic [1]. We also know that effective and timely treatment of women during pregnancy and labour significantly reduces perinatal transmission of HIV. ARV prophylaxis in infants further minimizes the risk of HIV acquisition, in particular if combined with safe breastfeeding practices [2]. More recent data from randomized clinical trials have shown that circumcision reduces female-to-male HIV transmission by nearly 60% and that ARV prophylaxis, for example, oral pre-exposure prophylaxis with such drugs as tenofovir and emtricitabine or the same combination in a gel format for topical use, can substantially reduce HIV acquisition if used consistently [3-5].

Despite these impressive advancements, in many high-prevalence settings, the majority of people do not know their HIV status, and according to global estimates, a mere 36% of those eligible for treatment are currently enrolled in treatment programmes [6-8]. In sub-Saharan Africa, where the greatest majority of HIV-infected children live, only 26% are receiving ARV therapy (ART). Moreover, while the treatment needs continue to grow, for every person with HIV placed on treatment, two more get newly infected. New infections also occur due to poor coverage of prevention of mother to child transmission programmes, which reach only half of all HIV-positive, pregnant women [6]. In sub-Saharan Africa particularly, tuberculosis (TB) case notification rates have spiralled out of control as a result of co-infection with HIV, and, despite effective treatment and prevention, TB continues to be the most important cause of death in people living with HIV, both in the pre-ART era and now, in the era of ART [9].

There are numerous impediments to achieving full implementation of all proven strategies, and multifaceted programmatic challenges create roadblocks to providing universal access to HIV prevention, treatment and care; social injustice, economic inequalities and operational obstacles continue to interrupt the access to, and the continuum of care.

Stigma and discrimination remain major hurdles to HIV testing, and hand in hand with persistent use of outdated and centralized systems, which lag behind modern and more efficient technologies, result in delayed provision of test results and low retention in care.

Most of sub-Saharan Africa’s health systems were organized and geared historically towards the management of acute illnesses, and are not orientated to the long-term management of chronic diseases, in particular to the scale of chronic disease caused by HIV. As a result, these health systems may quickly become overwhelmed and lack the capacity to carry and support the great burden of a growing – and chronic – patient pool. Health care providers, often overstretched, continue to juggle increasing numbers of patients in urgent need of care with limited resources. The demands and expectations are not only growing on healthcare providers, but on all other cadres of the broader health system. For example, reliable drug forecasting and appropriate inventory management to secure an uninterrupted supply of medicines and other necessities are indispensable elements of a successful ART programme.

Meeting all the needs and overcoming all the obstacles costs money. Governments, including those with the political will to make a change, struggle with setting priorities: balancing demand for financial investment in health systems, securing ART programmes through bilateral and multilateral relations to meet the urgent needs while, at the same time, seeking long-term sustainable solutions. Controversies as to whether funding for HIV interventions has sidelined investment in other health sectors in the context of a dire financial climate has perhaps contributed to HIV donor fatigue, which may overlook the positive spin-off effect of HIV funding on the broader health infrastructure and health outcomes.

Challenges are many and multifaceted, and it is increasingly evident that innovative approaches need to be conceived to more effectively use the scarce and shrinking resources. There is also a need to effectively address the growing demand within a framework that protects human rights and provides access to services through a public health approach.

Testimony to this priority shift is the change in terminology found across the literature. Within the HIV literature, key words have mushroomed, such as economic efficiency, trade offs, decentralization, task shifting, task sharing, empowerment, community involvement, home-based care, point-of-care tests for diagnosis and monitoring purposes, treatment simplifications, patient self-management, health system strengthening, and mobile technology. There has been a surge of innovative approaches and technologies to address the obstacles that we have mentioned and to optimize the operation of HIV care delivery.

The emerging operational research and implementation sciences within the HIV field dedicates its efforts to investigating and identifying the best models to deliver and implement services in various settings, taking into consideration the cultural, economical and political context.

Operational research is characterized by a “systems orientation, or systems engineering, in which interdisciplinary research teams adapt scientific methods to large-scale problems that must be modeled” [10]. In other words, operational research aims to provide a sound, rational basis for how to maximize the use of limited resources. Xiong *et al* propose that operational research can improve global HIV outcomes by supporting decision making both at a policy level, e.g., resource allocation, healthcare workforce planning and infrastructure planning, and at an operational level, e.g., demand forecasting, supply chain design, service benchmarking and service integration, many of which necessitate robust and reliable data collection and reporting [11]. Ultimately, addressing the operational obstacles lie at the heart of a successful response to the HIV epidemic.

In this special issue, the *Journal of the International AIDS Society* aims to highlight some of the operational and programmatic challenges that are faced in sub-Saharan Africa today, where the largest population of people living with HIV resides and where health expenditure and health infrastructure often fall short of the demands.

This supplement includes six articles on a diverse range of topics, from vital registries and reporting to various steps in programme delivery. We envisage that this supplement will serve to familiarize readers with a range of system matters encountered in the context of HIV clinical management in sub-Saharan Africa.

The first article in this supplement by Dlodlo *et al* shows the devastating impact of the HIV epidemic on the crude mortality in the two main cities of Zimbabwe (Harare and Bulawayo) due to a substantial increase in AIDS-related mortality. The authors further demonstrate how turning that tide is a challenging task; despite a 56% ART coverage, the crude mortality in Zimbabwe has declined by merely 19%. The paper highlights the importance of data collection and the value of using traditional data collection tools (the city council registries) that have withstood the test of time and the hugely changing economic circumstances that have occurred over the 30 years of this data collection study. Moreover, the paper shows how useful vital registries are in identifying the underlying factors and causes for increased mortality and morbidity, as well as how such data can be used to measure the impact of programmes at a population level.

The availability of human resources in primary healthcare and associated general health outcomes is investigated by Rasschaert and colleagues. This article provides examples from Malawi and Ethiopia. These countries, though different in size and population, have similar rankings on the Human Development Index, and both suffer from weak health infrastructures, shortages in the health workforce and low per capita health expenditure. The authors demonstrate how changes in human resources policies and addressing those shortcoming through task shifting and investment in functional health care facilities have had a beneficial impact on the broader health outcome in those countries as observed by some indicators, e.g., an increase in antenatal clinic attendance, a higher proportion of deliveries at health facilities, measles immunizations, and declines in maternal and under-five mortality.

Optimizing supply chain management has historically been a key area of focus of the operational research community. Schouten *et al* share their experiences in establishing a national ARV procurement mechanism in a changing environment in Malawi. Initially fairly straight forward with a single first-line ARV regimen, the drug forecasting and procurement system has become more complicated with increasing numbers of different regimens (e.g., alternative first line, second line and paediatric) and increasing volumes of drugs. The authors highlight that management of supply of various volumes of different ARV drugs requires a rethinking and a need for a dedicated forecasting and procurement team, a national buffer stock, storehouse capacity and a streamlining of the grant disbursement mechanism; these are issues that are also relevant to other sub-Saharan African contexts.

The daunting challenge of preventing, treating and managing TB among people living with HIV is described by Sculier *et al*. Despite effective measures to prevent, detect and treat TB, programmatic challenges result in TB remaining the leading cause of death in people living with HIV. In their paper, Sculier and co-authors provide an overview of available evidence and tools to scale up and integrate TB prevention and treatment with HIV programmes and improve the management of HIV-TB co-infections.

Reduction in viral load following effective treatment can substantially reduce the risk of HIV transmission. Early and widespread use of treatment, if implemented, is hypothesized to have a substantial impact on the HIV incidence in a community. The gold standard for testing the efficacy of this intervention can be provided by community randomized controlled trials. However, there remains a great deal of operational challenges to conducting such trials, and these are discussed by Williams *et al*. In their article, Williams *et al* examine the theoretical operational issues that need to be addressed, the obstacles to be overcome and the strategies that will need to be implemented for the community randomized trials to be successful in providing adequate evidence for the concept of treatment as prevention.

Finally, in a commentary with a futuristic perspective, Zachariah *et al* call for strengthening the operational research capacity and urge for accelerated research and proactive planning. The authors outline some of the current obstacles in reaching universal access in sub-Saharan Africa, and propose approaches that can considerably change the pace and make an impact on this epidemic. Key challenges for the future include: sustainability of funding for scale up; managing an ever-growing cohort on ART; getting those waiting for ART onto treatment; monitoring cohorts and ensuring long-term adherence; and investing in HIV prevention. Building capacity for operational research in order to provide answers to these challenges is urgently required.

From this special issue, we hope that readers may gain insight into some of the operational and programmatic challenges that are associated with the HIV/AIDS epidemic and a changing environment in sub-Saharan Africa, and become familiar with some applications of operational research and implementation science in HIV healthcare settings. The *Journal of International AIDS Society* accepts submissions in all disciplines and is particularly committed to prioritizing publications of operational research articles. We therefore strongly encourage submissions in this area and warmly welcome contributions that present innovative operational models or techniques that can help advance the scale up and optimization of care delivery in high-HIV-prevalence settings.
